# Relationships of capsular polysaccharides belonging to *Campylobacter jejuni* HS1 serotype complex

**DOI:** 10.1371/journal.pone.0247305

**Published:** 2021-02-23

**Authors:** Mario A. Monteiro, Yu-Han Chen, Zuchao Ma, Cheryl P. Ewing, Nooraisyah Mohamad Nor, Eman Omari, Ellen Song, Pawel Gabryelski, Patricia Guerry, Frédéric Poly

**Affiliations:** 1 Dept. of Chemistry, University of Guelph, Guelph, Ontario, Canada; 2 Naval Medical Research Center, Silver Spring, Maryland, United States of America; Cornell University, UNITED STATES

## Abstract

The *Campylobacter jejuni* capsule type HS1 complex is one of the most common serotypes identified worldwide, and consists of strains typing as HS1, HS1/44, HS44 and HS1/8. The capsule structure of the HS1 type strain was shown previously to be composed of teichoic-acid like glycerol-galactosyl phosphate repeats [4-)-α-D-Galp-(1–2)-Gro-(1-P-] with non-stoichiometric fructose branches at the C2 and C3 of Gal and non-stoichiometric methyl phosphoramidate (MeOPN) modifications on the C3 of the fructose. Here, we demonstrate that the capsule of an HS1/44 strain is identical to that of the type strain of HS1, and the capsule of HS1/8 is also identical to HS1, except for an additional site of MeOPN modification at C6 of Gal. The DNA sequence of the capsule locus of an HS44 strain included an insertion of 10 genes, and the strain expressed two capsules, one identical to the HS1 type strain, but with no fructose branches, and another composed of heptoses and MeOPN. We also characterize a HS1 capsule biosynthesis gene, HS1.08, as a fructose transferase responsible for the attachment of the β-D-fructofuranoses residues at C2 and C3 of the Gal unit. In summary, the common component of all members of the HS1 complex is the teichoic-acid like backbone that is likely responsible for the observed sero-cross reactivity.

## Introduction

*Campylobacter* is the main bacterial cause of bacterial foodborne disease in United States [1]. In low to middle income countries (LMIC), *C*. *jejuni* incidence is a major cause of moderate to severe diarrhea and, compared to adults, is estimated to be at least 10 times higher for children less than five years old [2]. Diarrhea induced by *C*. *jejuni* is often accompanied by fever, headache or myaglia [3]. In most cases the infection is self-limiting, but more severe cases require antibiotic treatment [3]. *C*. *jejuni* has also been linked to the development of post infectious sequelae including Guillain-Barré syndrome [4], Miller-Fisher Syndrome [5], irritable bowel syndrome (IBS) [6] and reactive arthritis [7,8]. In LMIC, *C*. *jejuni* infection has been linked to stunting in pediatric populations [9].

Characterization of *C*. *jejuni* pathogenesis factors has been hindered, in part, by the lack of small animal models mimicking human disease [10]. Only a few virulence factors have been identified, including the flagellum that is involved in motility and secretion of virulence proteins, the cytolethal distending toxin, the fibronectin binding protein cadF, the lipoprotein ceuE and the polysaccharide capsule (CPS). The *C*. *jejuni* CPS is composed of oligosaccharide repeats that commonly include heptoses in unusual configurations (e.g. *altro*, *gulo*), as well as *O*-methyl phosphoramidate (MeOPN) units on the sugars in serotype specific linkages [11]. The CPS of *C*. *jejuni* has been shown to be required for colonization of chickens [12] and mice [13], diarrhea in ferrets [14], and resistance to complement mediated killing [14,15]. Moreover, the CPS has been shown to be immunomodulatory [13,16].

*C*. *jejuni* CPS is the major determinant of the Penner or heat stable typing scheme, a passive slide hemagglutination assay that recognizes 47 different serotypes [17]. Recent development of a molecular CPS typing system re-enforced the strong correlation between CPS and Penner types [18,19]. Many Penner serotypes fall into related, cross-reacting complexes [19]. For example, in the HS23/36 complex strains can type as HS23, HS36 or HS23/36, but strains of each of these three serotypes have the same capsular gene content encoding proteins that are >97% identical [11]. HS23/36 is the only complex in which comparative structures of all members have been determined. The CPS structures of HS23, HS36 and HS23/36 strains [→ 3)-β-d-Glc*p*NAc-(1 → 3)-α-d-Gal*p*-(1→2)-6d-α-d-*alt*-Hep*p*-(1→] composed of trisaccharide repeating blocks of α-D-galactose, *N*-acetyl-β-D-glucosamine and 6-deoxy-3-*O*-methyl-α-D-*altro*-heptose (or its 3-*O*-methyl-L-*glycero*-α-D-*altro*-heptose variant) [11,20]. The CPS of an HS23/36 strain, 81–176, was shown to be modified non-stoichiometrically with MeOPN at three different positions (*O*-2, *O*-4 and *O*-6) on galactose [21].

We have explored the use of *C*. *jejuni* CPS conjugated to protein carriers as vaccine candidates, and have shown that an HS23/36-CRM197 conjugate provided 100% protection against diarrheal disease in non-human primates [22]. Both Penner serotyping and molecular CPS typing have revealed the predominance of 8–10 CPS types worldwide [23–26], and, thus, a final effective conjugate vaccine against *C*. *jejuni* should be multi-valent. The HS1 complex is one of the most common, accounting for ~8.2% of *C*. *jejuni* induced diarrhea worldwide [17,18]. Strains with this complex can serotype as HS1, HS44 or HS1/44. Recently, a highly virulent clone of *C*. *jejuni*, termed SA (Sheep Abortion), has replaced *Campylobacter fetus* as the main cause of ovine abortion in USA [27], and this clone has also been implicated as a cause of human gastroenteritis in the U.S. [28]. This SA strain types as a unique combination of serotype HS1/8 by the Penner serotyping system. Moreover, a specific allele of the *porA* gene, encoding the major outer membrane protein, has been shown to be critical for hypervirulence in animals [29], and another study has demonstrated that the presence of CPS is also critical [27].

**Fig 1:**
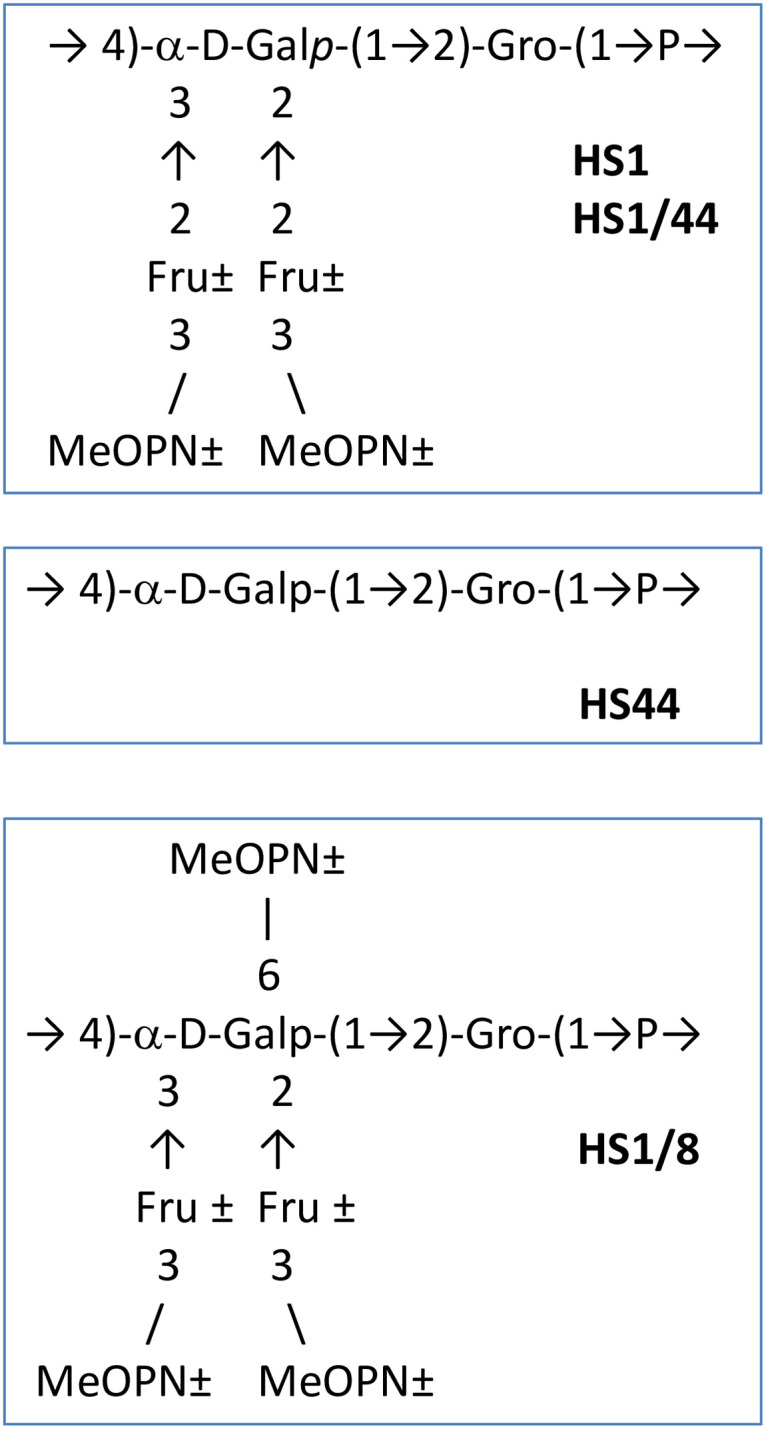
Structure of teichoic acid-like capsules among the HS1 complex.

**Fig 2:**
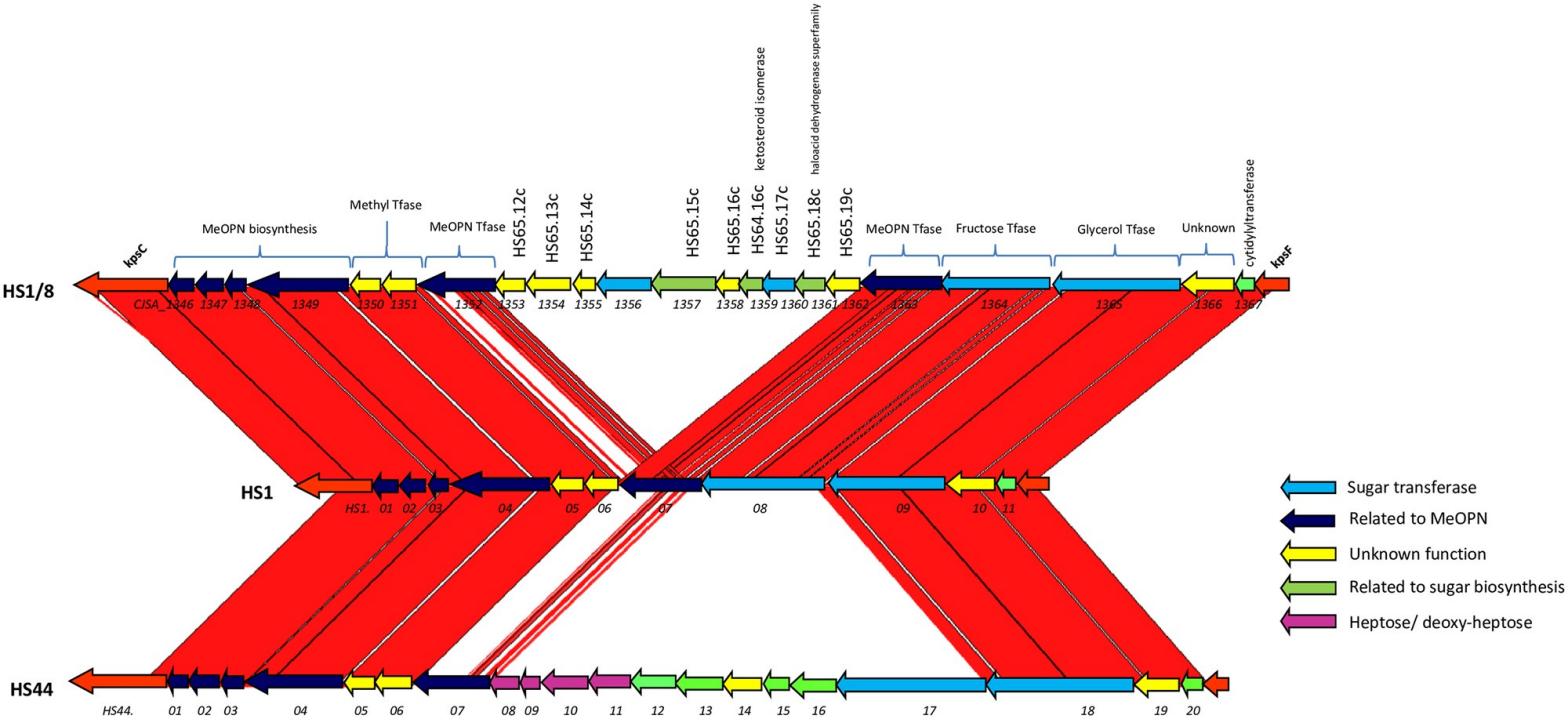
Alignment of variable CPS loci from *C*. *jejuni* HS1 and HS44 Penner type strains. Genes are color coded as follows: Navy blue, MeOPN biosynthesis and transferase; red, CPS transport and assembly; yellow, putative methyl transferase; purple, Heptose/deoxyheptose biosynthesis; blue, putative glycosyl transferase; green, sugar biosynthesis; brown, hypothetical.

The first structural studies on HS1 type-strain CPS (ATCC 43429) identified a linear teichoic-acid like CPS [→4)-α-D-Gal*p*-(1→2)-D-Gro-(1-P→]_n_ [30] (Fig 1). Further analyses confirmed the aforementioned teichoic-acid backbone sequence, but also revealed the presence of β-D-fructofuranoses (Fru) branches at C-2 and C-3 of the Gal unit, which in turn may be attached at C-3 with MeOPN [11]. Both fructofuranose branches and MeOPN are found in non-stoichiometric amounts on the HS1 CPS [31]. The ~15 kb HS1 CPS locus encoding eleven genes for the synthesis of this polysaccharide (GenBank accession number BX545859) is the smallest CPS locus identified to date in *C*. *jejuni* [11] (Fig 2). Since any multi-valent *C*. *jejuni* conjugate vaccine would have to include a representative or representatives from the HS1 complex, we have examined the relationship of the CPS structures of strains that serotype as HS1/44, HS44 and HS1/8 to that of HS1. We also examine the role of these CPSs in serum resistance.

## Materials and methods

### Bacterial strains and growth conditions

The strains used in this study are listed in Table 1. *C*. *jejuni* strains were routinely cultured at 37°C under microaerobic conditions (5% O_2_, 10% CO_2_, and 85% N_2_) on Mueller Hinton (MH) agar plates, supplemented with the appropriate antibiotic, if required. *E*. *coli* DH5α was used for cloning experiments and was grown in LB media supplemented with the appropriate antibiotics.

**Table 1:** Strains used in this work.

Strain no.	Strain name	Genotype/Relevant characteristics	Source
856	MSC57360	Penner type strain of HS1	J. Penner
2868		MSC57360 *kpsM*::*cat*	This work
3439		MSC57360 ΔHS1.08::*cat*	This work
3508		MSC57360 ΔHS1.08::*cat*, pCPE28 + HS1.08	This work
3361		MSC57360 *mpnC*::*cat*	This work
2871	ATCC 43463	Penner type strain of HS44	ATCC
2889		ATCC 43463 *kpsM*::*cat*	This work
3451		ATCC 43463 *mpnC*::*cat*	This work
3087	CG98-U-77	Thai clinical isolate; HS1/44	C. Mason
3352	IA3902	Sheep abortion isolate; HS1/8,	Q. Zhang

### DNA purification and CPS sequencing strategies

*C*. *jejuni* genomic DNA was extracted from 16 hour cultures following the method described by Sambrook et al. [32]. Sequencing of the CPS loci was performed as previously described [11,18,33]

### Extraction, purification and structural analysis of water-soluble CPS material

The CPS was extracted from cells by hot water–phenol extraction for 2 h at 70°C. The aqueous layer was dialyzed (1000 Da) against water followed by ultracentrifugation at 40,000 r.p.m. (Beckmann Type 45 Fixed-Angle Rotor) for 5 hrs at 4°C to separate the CPS from the LOS. The supernatant material containing was subjected to size-exclusion chromatography (Sephadex G50) for further purification to yield CPSs. Monosaccharide composition was performed using a procedure amenable to the alditol acetate method [34] with the alditol acetates being analyzed in a Thermo Finnigan Polaris-Q gas chromatograph/mass spectrometer (GC/MS) using a DB-17 capillary column. The sugar linkage types were characterized by characterization of the permethylated alditol acetates by GC/MS as previously described [34]. The NMR experiments were performed on a Bruker 400 MHz spectrometer equipped with a Bruker cryo platform at 295 K. The 1D and 2D NMR experiments were performed using Bruker standard software. For the ^1^H and ^13^C NMRs deuterated trimethylsilyl propanoic acid was used as the external reference and orthophosphoric acid was the external reference.used to calibrate the ^31^P NMR experiments.

### PCR primers

The primers were designed using the Primer3 software (http://bioinfo.ut.ee/primer3-0.4.0/). All primers were synthesized by ThermoFisher Scientific custom DNA oligos synthesis services and are listed in Table 2.

**Table 2:** Primers used in this work.

Primer name	Primer sequence (5’-3’)
HS1.08_BamHI_F	ATCGGGATCCATGGACATGCAAGAACCTTT
HS1.08_XhoI_R	ATCGCTCGAGTGCAACTCTTTAGCAACATTGG
HS1.08_EcoRI_F	ATCGGAATTCAAATATAGAGCAAGGTGTTCAAAAA
HS1.08_PstI_R	ATGCCTGCAGTCAGTTGCGCTAAAATTAAGAAAA
CmR_EcoRI_F	ATCGGAATTCTCGGGGCGGTGTTCCTTTCCAAG
CmR_ PstI_R	ATGCCTGCAGGCCCTTTAGTTCCTAAAGGGT
pg12.60	CGGGATCCGTTTAGAGAGGATTAAAAATGTTTTTTTTG
pg12.61	GGAATTCCATCTCCATAAAATAAAACAGCAACTC

### Mutational analyses

The HS1.08 gene was amplified by PCR from the *C*. *jejuni* HS1 type strain using the following primers: HS1.08_BamHI_F, HS1.08_XhoI_R. The PCR product was cloned into the BamHI and XhoI restriction site of pBluescript KS(+) (Stratagene). Ligations were transformed into *E*. *coli* DH5α and plated on LB agar plates supplemented with ampicilin and X-gal. White colonies were selected and confirmed to contain the insert of the correct size by PCR using the same primers used for cloning. A positive clone for each construct was use as a template in an inverse PCR reaction in conjunction with HS1.08_EcoRI_F and HS1.08_PstI_R to confirm a deletion of 1321bp. The chloramphenicol (Cm^R^) resistance cassette was amplified from pUOA18 DNA [35] with primers CmR_EcoRI_F and CmR_ PstI_R. PCR fragments and purified plasmids were digested with EcoRI and PstI enzymes, ligated and cloned into *E*. *coli* DH5α. Selection of clones containing ΔHS1.08 Cm^R^ was performed by PCR using HS1.08_BamHI_F/HS1.08_XhoI_R. A positive clone was selected and their plasmids purified and used to electroporate *C*. *jejuni* strains as previously described [10]. The mutant was termed 3439 (see Table 1).

Non-encapsulated mutants of HS1 (strain 2868) and HS44 (strain 2889) were constructed by electroporation of plasmid pDB173 encoding *kpsM* of *C*. *jejuni* 81–176 insertionally inactivated with a kanamycin (Km^r^) resistance gene [14].

Mutants of HS1 (strain 3361) and HS44 (strain 3451) that were unable to synthesize MeOPN were constructed by electroporation of a plasmid containing the *mpnC*::*cat* allele previously described [13] into each strain. In all cases mutations were confirmed by PCR analyses using primers that bracketed the insertion point of the antibiotic resistance cassette used.

### Complementation of HS1.08 in trans

The HS1.08 gene was PCR amplified from HS1 with Amplitaq with primers pg12.60 and pg12.61. The primer pair introduced EcoRI and BamHI restriction sites at each end, and the amplicons were digested with these enzymes and cloned into EcoR1-BamHI digested pCPE28, which is the kanamycin resistance (Km^r^) campylobacter shuttle vector pRY107 with a sigma^28^ promoter cloned between the XbaI and BamHI sites in the polylinker [36,37]. Appropriate plasmids were transformed into DH5α cells carrying the conjugative plasmid RK212.2 as previously described [38,39]. The resulting cells were used as donors to transfer the complementing plasmid conjugatively into the *C*. *jejuni* HS1 HS1.08 mutant (strain 3439) with selection for Km^r^ as previously described [38,39].

### SDS-PAGE analyses

Crude capsule preparations were prepared for SDS-PAGE analyses by proteinase K digestion of whole campylobacter cells as previously described [23]. Following electrophoresis on 12.5% SDS-PAGE gels, gels were washed twice in water for 20 min and stained for 30 minutes in 0.5% Alcian blue, 2% acetic acid solution at room temperature. De-staining was performed in 2% acetic acid solution at room temperature overnight [24].

### Complement killing

Pooled normal human sera (NHS) was purchased from Sigma and a single lot used for all experiments. Assays were done as described in Pequegnat et al., [21] using a range of NHS. Assays were repeated between 3–5 times for each strain. Statistics were done using GraphPad Prism.

### Accession numbers

The CPS sequence of ATCC 43463 (HS44) described in this paper has been submitted to GenBank under accession number JF496678.

## Results

### Sequence comparison of CPS loci within the HS1 complex

The genetic organization of the CPS genes of *C*. *jejuni* is similar to Class 2 and Class 3 CPS loci of *E*. *coli*. Thus, the variable region containing the genes for synthesis of the polysaccharide are located between the conserved genes encoding the ABC transporter involved in capsule synthesis and assembly. The variable region of the HS1 CPS locus is shown in Fig 2 and the genes are listed in Table 3 [31]. The DNA sequence of the capsule locus of the HS44 type strain contained homologs of 10 of the 11 genes found in HS1, missing only a homolog of HS1.08, a gene of unknown function (Fig 2). All shared homologs were >96% identical, except for the putative MeOPN transferase (HS44.07) which showed only 47% identity to that of HS1. The HS44 locus included an insertion of 10 additional genes between HS1.07 and HS1.09 encompassing 9,258 bp (Table 3, Fig 2). These include genes having >96% homology to *C*. *jejuni* genes encoding enzymes predicted to be involved in heptose/deoxyheptose biosynthesis (HS44.08 to HS44.11) and three genes (HS44.12, HS44.13 and HS44.15) encoding proteins that are homologous to epimerase reductases that have been recently suggested to be also involved in heptose/deoxyheptose sugar modification [25]. The CPS locus of HS44 also includes a gene (HS44.14) similar to Cj1429c coding for a protein of unknown function in *C*. *jejuni* strain NCTC 11168 (HS2), a putative nucleotidyl-sugar pyranose mutase (HS44.16) and a putative heptosyltansferase (HS44.17, Table 3 and Fig 2).

**Table 3:** Comparison of gene content of HS1 and HS44 capsule biosynthesis loci.

Locus Tag	Putative function^a^	Relationship	Identity with HS1^b^	Size (amino acid)
HS44.01	MeOPN biosynthesis	HS1.01	164/170 (96%)	170
HS44.02	MeOPN biosynthesis	HS1.02	252/253 (99%)	253
HS44.03	MeOPN biosynthesis	HS1.03	197/200 (98%)	200
HS44.04	MeOPN biosynthesis	HS1.04	775/779 (99%)	779
HS44.05	Methyl transferase	HS1.05	253/253 (100%)	253
HS44.06	Methyl transferase	HS1.06	255/257 (99%)	257
HS44.07	MeOPN transferase	HS1.07	308/642 (47%)	609
HS44.08	sugar-phosphate nucleotidyltransferase	-	-	224
HS44.09	sedoheptulose 7-phosphate isomerase	-	-	201
HS44.10	D-glycero-D-manno-heptose 7-phosphate kinase	-	-	360
HS44.11	GDP-mannose 4,6-dehydratase	-	-	343
HS44.12	GDP-fucose synthetase (fcl)	-	-	381
HS44.13	GDP-fucose synthetase (fcl)	-	-	385
HS44.14	*Cj1429* like	-	-	310
HS44.15	Nucleotide-sugar epimerase/dehydratase	-	-	181
HS44.16	Nucleotidyl-sugar pyranose mutase	-	-	416
HS44.17	Heptosyl transferase	-	-	1202
HS44.18	CDP glycerol glycerophosphotransferase	HS1.09	1067/1095 (97%)	1100
HS44.19	Unknown	HS1.10	390/396 (98%)	397
HS44.20	Glycerol-3-phosphate cytidylyltransferase	HS1.11	128/129 (99%)	129

^(a)^ Function attributed based on Blastp performed on non-redundant protein sequences database.

^(b)^ numbers In parenthesis are the percentage of identity between the HS1 and HS44 proteins.

The sequence of the capsule locus of the HS1/8 IA3902 SA strain was recently published [27]. The HS1/8 CPS biosynthesis locus contains all 11 of the genes present in the HS1 type strain, and it also contains an insertion located between HS1.06 and HS1.07 that is unrelated to the one seen in HS44 (Fig 2). The HS1/8 insertion contains 10 genes, 9 of which are highly conserved with the genes of the CPS locus of HS65, a member of the HS4 complex. The tenth gene, CJSA_1356, appears to be HS1/8 specific, and encodes for a predicted 639 amino acid protein with weak homology to a sugar transferase (Table 4). Interestingly, IA3902 contains two genes predicted to be MeOPN transferases: CJSA_1352 which is 67% identical to HS1.07 and CJSA_1363 which is 100% identical to HS1.07 (Table 4).

**Table 4:** Comparison of gene content between *C*. *jejuni* IA3902 and HS1 capsule biosynthesis locus.

Locus Tag	Putative function^a^	Gene name	Relationship	Identity with HS1^*b*^	Size (amino acid)
CJSA_1346	MeOPN biosynthesis	*mpnD*	HS1.01	169/170(99.41%)	170
CJSA_1347	MeOPN biosynthesis	*mpnC*	HS1.02	252/253(99.6%)	253
CJSA_1348	MeOPN biosynthesis	*mpnB*	HS1.03	196/200(98%)	200
CJSA_1349	MeOPN biosynthesis	*mpnA*	HS1.04	772/779(99.1%)	779
CJSA_1350	Methyl transferase	-	HS1.05	252/253(99.6%)	253
CJSA_1351	Methyl transferase	-	HS1.05	104/257(33.07%)	257
CJSA_1352	MeOPN transferase	-	HS1.07	434/641 (67%)	612
CJSA_1353	Unknown	-	-	-	240
CJSA_1354	Unknown	-	-	-	507
CJSA_1355	Unknown	-	-	-	132
CJSA_1356	Sugar transferase	-	-	-	639
CJSA_1357	dTDP-glucose pyrophosphorylase	-	-	-	619
CJSA_1358	Unknown	-	-	-	109
CJSA_1359	ketosteroid isomerase	-	-	-	111
CJSA_1360	glycosyl transferase	-	-	-	241
CJSA_1361	haloacid dehalogenase	-	-	-	219
CJSA_1362	Unknown	-	-	-	212
CJSA_1363	MeOPN transferase	-	HS1.07	631/631(100%)	637
CJSA_1364	Heptosyl transferase	*hddD*	HS1.08	830/830(99.4%)	851
CJSA_1365	CDP glycerol glycerophosphotransferase	*tagF*	HS1.09	1095/1095(100%)	1095
CJSA_1366	Unknown	-	HS1.10	402/402(100%)	402
CJSA_1367	Glycerol-3-phosphate cytidylyltransferase	*tagD*	HS1.11	129/129(100%)	129

^(a)^ Function attributed based on Blastp performed on non-redundant protein sequences.

In contrast, the DNA sequence of the variable CPS locus of a clinical isolate that typed as HS1/44 was identical with that of the type strain of HS1. The minimum protein homology predicted from the 11 genes in these two capsule loci was >99%.

### CPS structure of a *C*. *jejuni* HS1/44 Penner clinical isolate (strain 3087)

Monosaccharide composition analysis (via characterization of the alditol acetate derivatives by GC-MS) of *C*. *jejuni* serotype HS1/44 (strain 3087) CPS revealed the presence of glycerol (Gro) and galactose (Gal). Mannose (Man) and glucose (Glc) were also detected, which were later reasoned to have originated from fructose (Fru). Penner *C*. *jejuni* HS1 type strain (strain 856), used here as a structural reference point, afforded a similar CPS monosaccharide composition. The GC-MS profiles of the HS1/44 and HS1 alditol acetate mixtures showed the Gal derivative as the dominant peak. The peaks assigned to Gro, Man and Glc were of lower intensity. Monosaccharide linkage analysis (through characterization of permethylated alditol acetate derivatives by GC-MS) revealed that HS1/44 and Penner HS1 type strain CPSs contained 4-substituted [→4)-Gal*p*-(1→] and 2,3,4-trisubstituted Gal [→2,3,4)-Galp-(1→] units. Terminal Gal*p* was also detected in lesser amount. The glycose composition of HS1/44 CPS suggested that this serotype might indeed be similar in structure to that previously reported for *C*. *jejuni* HS1 type strain [30,31].

**Fig 3:**
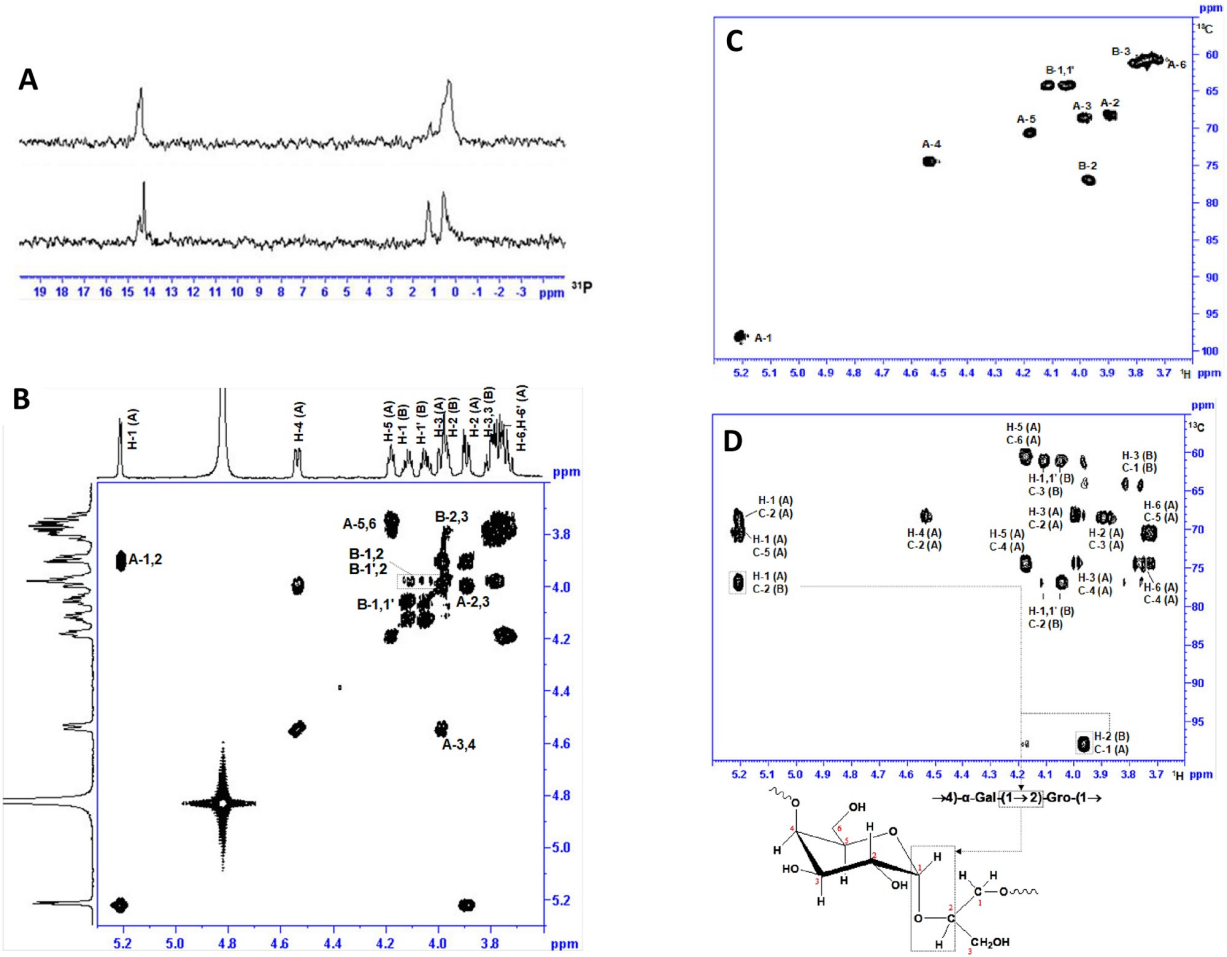
NMR of Penner HS1 type strain native CPS and HS1/44 native CPS and defructosylated CPS. (A) 1D ^31^P NMR spectra of *C*. *jejuni* Penner HS1 type strain CPS (top) and HS1/44 CPS (bottom) showing the teichoic-acid phosphate resonances (0.02–1.25 ppm) and the MeOPN-3-Fru resonances (14.25–14.85 ppm); (B) 2D ^1^H-^1^H COSY spectrum of defructosylated HS1/44 CPS; (C) 2D ^1^H-^13^C HSQC spectrum of defructosylated HS1/44 CPS; (D) 2D ^1^H-^13^C HMBC spectrum of defructosylated HS1/44 CPS.

Explorative 1D ^1^H and ^31^P NMR studies on HS1/44 CPS and Penner HS1 type strain furnished comparable resonance profiles, with resonances in the anomeric region, at δ 5.21 and δ5.38, later attributed to the 4-substituted Gal unit and 2,3,4- trisubstituted Gal, respectively. The relative intensities of the two α-Gal anomeric resonances at δ 5.21 and δ 5.38, pointed to the fact that HS1/44 CPS possessed a lesser number of 2,3,4- trisubstituted Gal than the Penner HS1 type strain. The ^1^D ^31^P NMR spectra (Fig 3A) of Penner HS1 type strain (top) and HS1/44 (bottom) both revealed two distinct phosphate resonance clusters, at δ 14.20–14.85 and at δ 0.02–1.25, the former would be later assigned to MeOPN moieties at Fru units and the latter to the CPS backbone teichoic-acid diesterphosphate.

2D ^1^H NMR experiments carried out on HS1/44 CPS yielded convoluted spectra that made it difficult to unambiguously assign all CPS proton resonances. To aid in the full assignment of 2D NMR data, the HS1/44 CPS, presumed to contain MeOPN-Fru side-branches, was defructosylated through the selective removal of the MeOPN-Fru side-branches through treatment with mild acid (1% acetic acid at 100 °C for 1hr). GC-MS sugar analysis of defructosylated HS1/44 CPS revealed only 4-substituted Gal and no 2,3,4-trisubstituted Gal. The 1D ^31^P NMR spectrum of defructosylated HS1/44 CPS also pointed to the fact that MeOPN substituents were no longer present, with only a phosphate resonance at δ 1.2 being observed. Through a 2D ^1^H-^1^H COSY NMR experiment (Fig 3B) on defructosylated HS1/44 CPS, the α-anomeric resonance (H-1) at δ 5.21 (*J*_1,2_ 3.8 Hz) was determined to be associated with resonances at δ 3.89 (H-2; *J*_2,3_ 10.5 Hz), δ 3.99 (H-3; *J*_3,4_ 2.8 Hz), δ 4.53 (H-4; *J*_4,5_ 2.4 Hz), 4.18 (H-5; *J*_5,6_ 6.0 Hz) and H-6,6’ (δ 3.74; m) and thus assigned to an α-Gal unit (residue A). Proton resonances (multiplets) belonging to Gro (residue B) emanated at δ 4.11/δ 4.05 (H-1/H-1’), δ 3.97 (H-2) and δ 3.78 (H-3/3’).

Using the data obtained from 2D ^1^H-^1^H COSY/TOCSY experiments, the carbon resonances of HS1/44 defructosylated CPS were assigned with a 2D ^1^H-^13^C HSQC NMR experiment (Fig 3C): δ 98.1 (C-1), δ 68.2 (C-2), δ 68.5 (C-3), δ 74.4 (C-4), δ 70.6 (C-5), δ 60.6 (C-6) for α-Gal unit; and for Gro at δ 64.3 (C-1), δ 77.1 (C-2) and δ 61.1 (C-3). Key data defining the inter-sugar linkages in the HS1/44 defructosylated CPS was afforded by a 2D ^1^H-^13^C HMBC NMR experiment (Fig 3B): H-1 of Gal (A) correlated with C-2 of Gro (δ_H_ 5.21/δ_C_ 76.9) and H-2 of Gro (B) with C-1 of Gal (δ_H_ 4.97/δ_C_ 97.9) for a Gal-(1→2)-Gro sequence. The cross-peaks observed at δ_H_ 4.53/δ_P_ 1.2 (H-4 of Gal correlation with phosphate) and δ_H_ 4.11,4.05/δ_P_ 1.2 (H-1 of Gro correlation with phosphate) in a 2D ^1^H-^31^P HMBC NMR spectrum locked in a Gro-(1→P→4)-Gal sequence. Collectively, the aforementioned structural data showed that the *C*. *jejuni* HS1/44 CPS is composed of a teichoic-acid backbone: [→2)-Gro-(1→P→4)-Gal-(1→], similar to that in HS1 type strain ATCC 43429 [30,31]. No other glycans were detected in the defructosylated HS1/44 CPS preparation.

**Fig 4:**
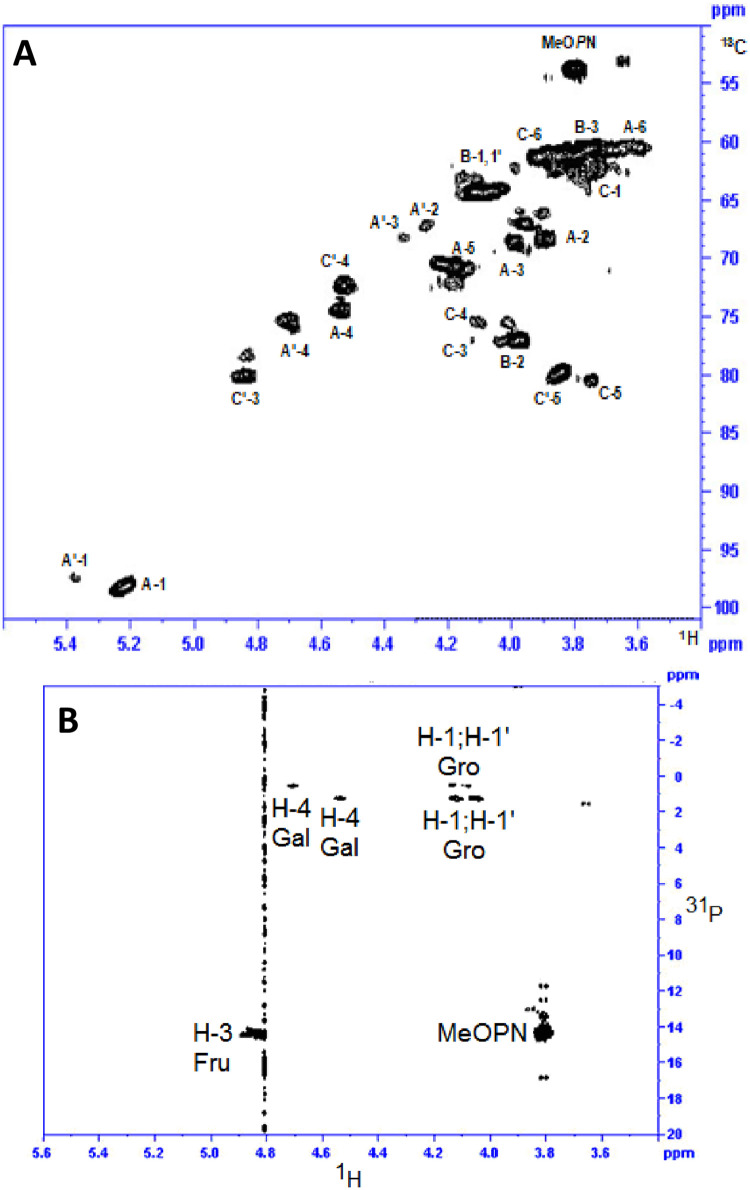
NMR of HS1/44 CPS. (A) 2D ^1^H-^13^C HSQC spectrum of HS1/44 native CPS; (B) 2D ^1^H-^31^P HMBC spectrum of HS1/44 native CPS.

The assignment of the proton and carbon resonances of the defructosylated HS1/44 CPS [→2)-Gro-(1→P→4)-Gal-(1→], now aided in the interpretation of more complex 2D NMR spectra generated by native HS1/44 CPS. A 2D ^1^H-^1^H COSY NMR spectrum of HS1/44 CPS allowed the assignment of proton resonances belonging to the 2,3,4-Gal substituted with Fru at C-2 and C-3 positions (residue A’): H-1 (δ 5.38), H-2 (δ 4.27), H-3 (δ 4.34), H-4 (δ 4.71), H-5 (δ 4.18) and H-6,6’ (δ 3.74) (Table 5). The proton resonances of the trisubstituted Gal (residue A’) were observed to resonate slightly downfield to those of residue A (monosubstituted 4-Gal). With fresh knowledge about proton resonance chemical shifts, the carbon resonances of HS1/44 CPS were determined through a 1D ^1^H-^13^ C HSQC NMR (Table 5; Fig 4A).

**Table 5:** ^1^H and ^13^C NMR chemical shifts of *C*. *jejuni* HS1/44 CPS.

Residue	H-1/C-1	H-2/C-2	H-3/C-3	H-4/C-4	H-5/C-5	H-6/C-6	CH_3_
2,3,4-Gal (A’)	5.38/97.5	4.27/67.2	4.34/68.3	4.71/75.3	4.18/70.6	3.74/60.6	
4-Gal (A)	5.22/97.1	3.90/68.5	3.99/69.0	4.52/75.2	4.38/72.5	3.63/60.6	
3-Fru (C’)	3.63,3.74/62.2	-/103.4	4.84/80.1	4.53/72.3	3.84/79.7	3.90,3.77/61.2	
Fru (C)	3.63,3.74/62.2	-/103.4	4.12/77.0	4.11/75.4	3.75/80.3	3.90,3.77/61.2	
Gro	4.05,4.15/64.5	3.98/77.5	3.78/60.5				
MeOPN							3.81/53.9

Two methylene resonances were observed in the 2D ^1^H-^13^C HSQC/HMBC NMR (Fig 4A) and assigned to positions 1 and 6 of Fru units (δH 3.63, δH 3.74/δC 62.2) and (δH 3.90, δH 3.77/δC 61.2), respectively. Two distinct Fru ring systems were observed, which were label C (Fru units without MeOPN) and C’ (Fru units with MeOPN at C-3) as evaluated by the H-3/C-3 resonances of C and C’, with that of C’ MeOPN (δ_H_ 4.83) being associated with MeOPN (δ_P_ 14.3) in a 2D ^1^H-^31^P HMBC experiment (Fig 4B). Fig 4B also showed a correlation between the diesterphosphate (δ_P_ 0.5 and 1.5) and H-1,1’ of Gro units and H-4 protons of 4-linked Gal (A) and 2,3,4-linked Gal (A’) emanating from the CPS teichoic acid backbone.

### CPS structure of *C*. *jejuni* HS44 (strain 2871)

**Fig 5:**
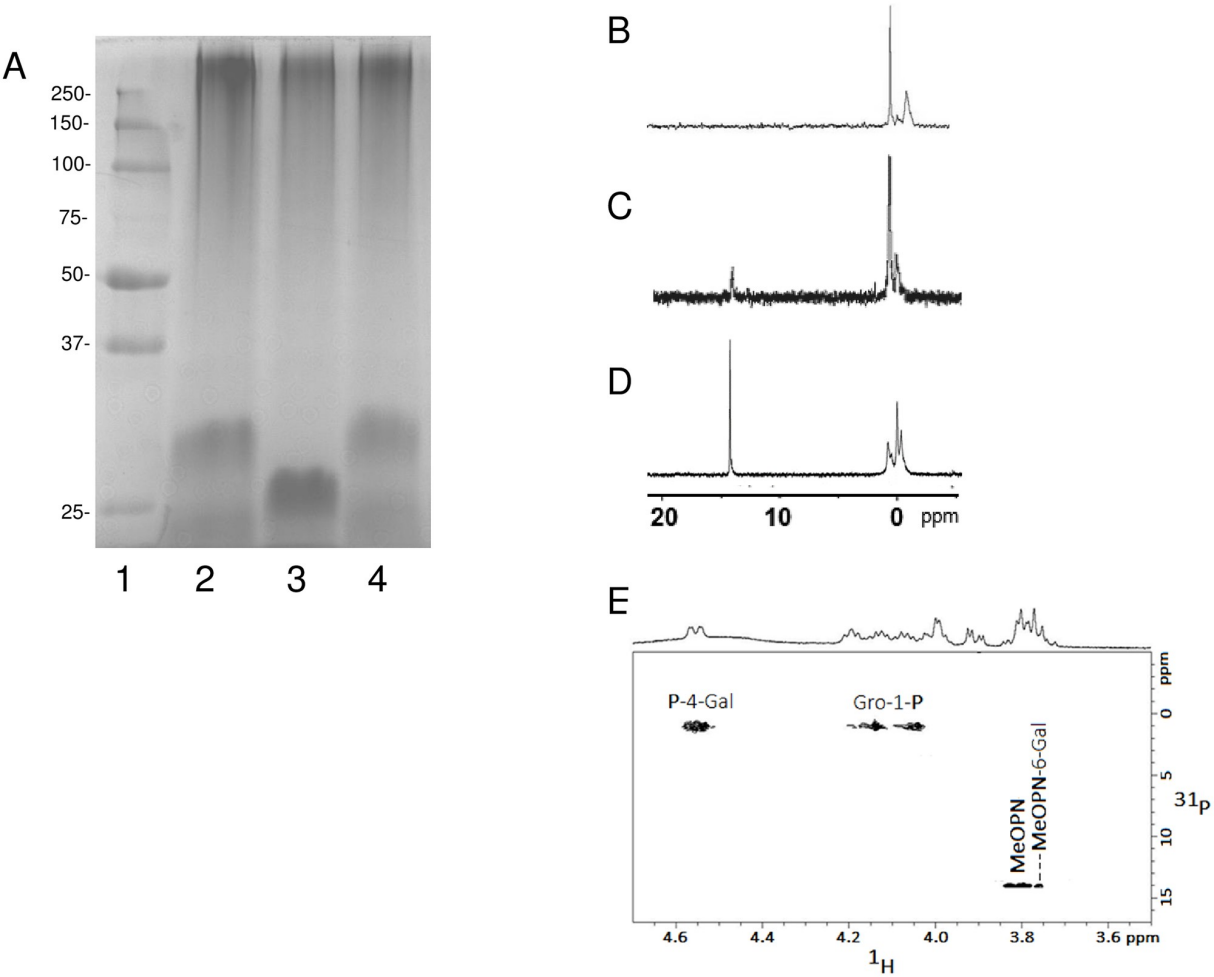
Characterization of mutants in the HS1.08 gene. A. Alcian blue stained 12.5% SDS PAGE of crude CPS preparations. Lane 1, Precision Plus protein standards; lane 2, HS1 wildtype; lane 3, HS1 1.08 mutant; lane 4, HS1 1.08 mutant complemented; B. ^31^P NMR of CPS from strain HS1 ΔHS1.08::cat showing the CPS devoid of MeOPN; C. ^31^P NMR spectrum of CPS from HS1 1.08 mutant complemented, showing the re-insertion of MeOPN in the CPS.; D. 1D ^31^P NMR of *C*. *jejuni* HS44 (strain 2871) CPS preparation; E. ^1^H-^31^P HMBC NMR of defructosylated *C*. *jejuni* HS1 strain 3588.

Monosaccharide composition and linkage analysis of HS44 CPS material HS44 CPS showed the presence of of Gro and 4-substituted Gal as found in the teichoic-acid CPS backbone of HS1 and HS1/44, but no Man units (that would have originated from NaBH_4_ reduction of Fru residues was observed (Fig 1). No Fru ^1^H or ^13^C resonances were detected in the NMR experiments of HS44 CPS material Consistent with the gene insertions described above, the CPS material was rich in heptoses, 6-deoxy-*galacto*-heptose (6d-*gal*-Hep), 6-deoxy-*altro*-heptose (6d-*altro*-Hep) and, in lesser amounts, 6-deoxy-3-*O*-methyl-*altro*-heptose (6d-*altro*-3-*O*-Me-Hep). The heptose configurations were characterized by comparison with well-defined synthetic standards by GC. A 1D ^31^P NMR spectrum showed the HS1 characteristic teichoic-acid phosphate (δ_P_ -0.02 to 0.99), but also a new MeOPN moiety (δ_P_ 14.05), distinct from the MeOPN expressed by HS1 and HS1/44, consistent with the divergence of the putative MeOPN transferase (HS44.07) observed in this strain (Fig 5D). Preliminary data pointed to the fact that HS44 contains components of HS1 teichoic-acid CPS, (devoid of fructose branches) and another CPS with deoxy-heptose constituents, as found in other *C*. *jejuni* serotypes [11]. The fine structure of HS44 CPS constituents will be published at a future date.

### CPS structure of *C*. *jejuni* HS1/8 (strain IA3902; 3352)

A structural examination revealed that the CPS of HS1/8 (IA3902) was similar to the CPSs of Penner HS1 type strain and HS1/44 strain, but with an additional MeOPN residue at C-6 of Gal (Fig 1) characterized by a correlation between a MeOPN resonance at δ_P_ 14.15 and position 6 (δ_H_ 3.75) of Gal. Fig 5E displays a 2D ^1^H-^31^P NMR spectrum of the defructosylated CPS of a related HS1 strain (3588) that shows the correlations between the P and Gal/Gro units of the teichoic-acid backbone (Fig 1) and that of the side-branch MeOPN attached to the 6-position of the backbone Gal. Previously, this MeOPN-6-Gal linkage has also been immunochemically observed in the CPS of HS23 serotype, through reaction of HS23 CPS with sera raised against a synthetic MeOPN-6-Gal conjugate [40]. In this study, there was no evidence of a second CPS in strain IA3902, despite the insertions within the CPS locus.

### Mutational analysis of HS1 CPS genes

The product of the HS1.08 gene encodes a predicted protein of 849 amino acids that was annotated as a putative sugar transferase [11]. Because the HS44 teichoic acid-like CPS lacked the non-stoichiometric fructose branch (Fig 1) and the HS1.08 gene was missing from the capsule locus, we hypothesized that HS1.08 encoded a fructose transferase. The HS1, HS1.08 mutant resulted in a CPS devoid of MeOPN-Fru side branches (Fig 5B). The mutant in this gene in HS1, strain 3439, expressed a lower MW capsule as on an Alcian blue stained gel and the MW was restored to that of wildtype in the complement, strain 3508, as shown by gel and NMR analysis (Figs 3A and 5A top panel). Complementation of the HS1.08 mutant in strain 3508 restored the presence of MeOPN and Fru, but the lower intensity of the MeOPN resonance in the ^31^P NMR (Fig 5C) suggested that complementation in this case was partial. Thus, HS1.08 appears to encode a transferase responsible for the transfer of Fru to Gal.

### The role of MeOPN in serum resistance in the HS1 complex

**Fig 6:**
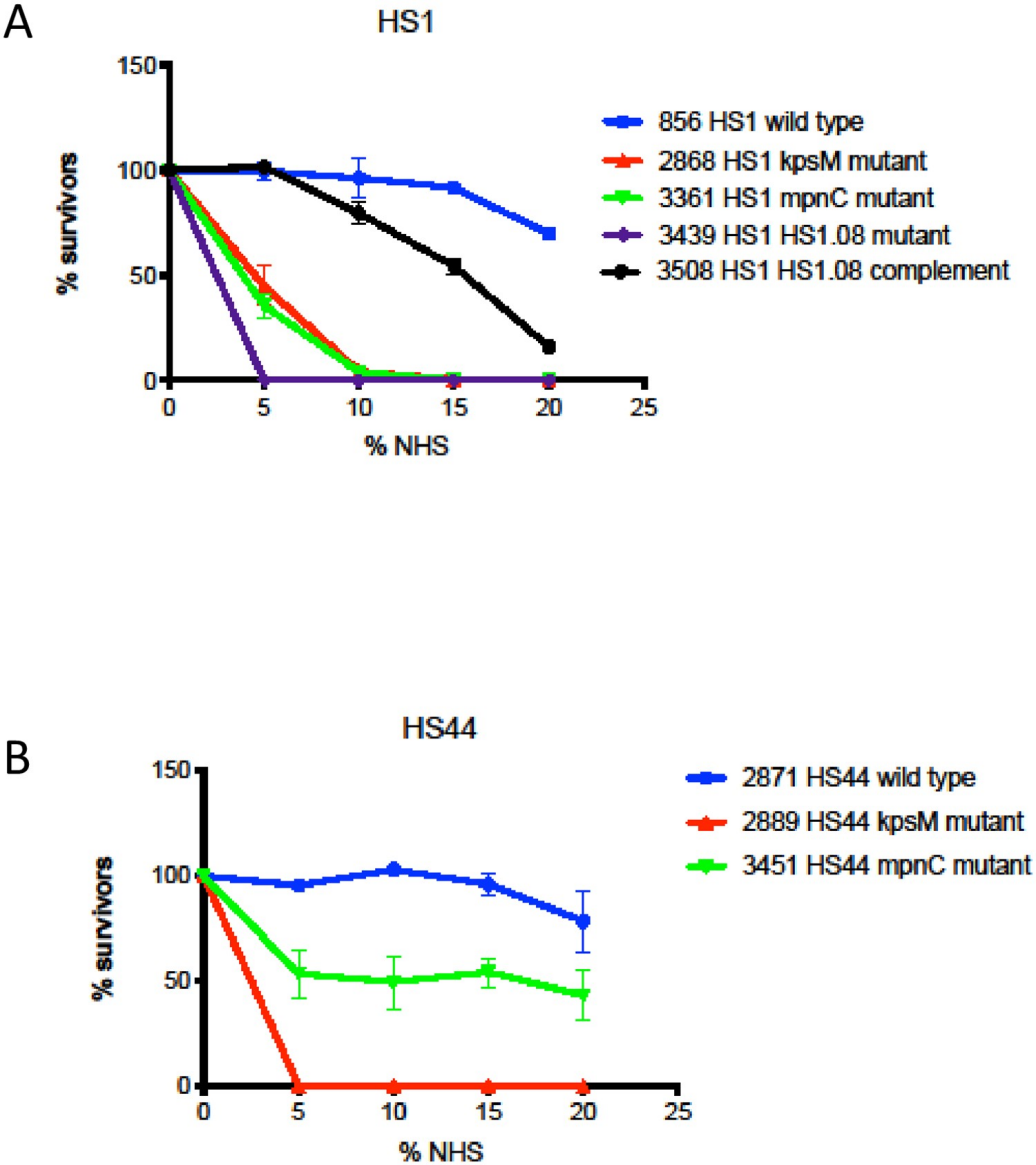
Bacterial sensitivities to complement killing by Normal Human Serum (NHS) serum killing. (A) *C*. *jejuni* HS1 strain and mutants, (B) *C*. *jejuni* HS44 strain and mutants.

**Since** we have previously shown that MeOPN is critical to serum resistance in HS23/36 strains (22), we examined complement resistance in representative strains of the HS1 complex, as shown in Fig 6. The wildtype HS1 strain showed high levels of resistance at all levels of NHS tested. Similar to what has been described for HS23/36, a mutant in *mpnC*, which is unable to synthesize MeOPN (green line) and a mutant lacking all CPS (red line) were significantly more sensitive to killing than wildtype even at 5% NHS (<0.05). The HS1.08 mutant in HS1, strain 3439, only harbor the techoid-acid like backbone as CPS. Complement resistance analysis show that the isogenic mutant is sensitive like the HS1 *mpnC* mutant. Complementation, strain 3508 restore partially serum resistance.

We also examined complement killing in the HS44 genetic background in Fig 6B. The wildtype strain was resistant to serum killing at all levels of NHS tested (blue line) and the *kpsM* mutant (red line), strain 2889, was significantly less resistant to killing at all levels of NHS. However, the *mpnC* mutant, strain 3451, showed an intermediate level of resistance that did not reach significance compared to wildtype.

## Discussion

This is the first study of the structures of CPS of multiple members of the HS1 complex. The structure of HS1 type strain ATCC 43429 has been previously described as a teichoic acid like structure composed of [4-)-α-D-Galp-(1–2)-Gro-(1-P-] repeats with non-stoichiometric fructose branches at the C2 and C3 of Gal and non-stoichiometric MeOPN modifications on the C3 of fructose [11]. A clinical isolate that typed as HS1/44 and a sheep abortion isolate that typed as HS1/8 expressed a similar CPS structure, although the levels of fructose and MeOPN varied in the populations examined. Preliminary analysis on strain of HS44 also pointed to the same teichoic acid like repeating structure but lacked the fructose branches (and attached MeOPN) due to loss of a gene identified in this study as the fructose transferase (HS1.08). The fructose transferase in HS44 was replaced by an insertion of 9 genes most of which encoded predicted proteins that are similar to enzymes involved in heptose synthesis (HS44.08-HS44.17; see Table 3) and a gene homologous to a MeOPN transferase (HS44.07). This is consistent with the presence of a second, heptose containing CPS in this strain. Interestingly, the HS1/8 strain, IA3902, was previously shown to contain a distinct insertion within the HS1-like CPS locus, but no other CPS was observed in the HS1/8 strain under the conditions of growth used in this study.

Here we have established that HS1.08 encodes the fructose transferase. Although the fructose branches on HS1 CPS are non-stoichiometric, HS1.08 lacks obvious homopolymeric tract that would be responsible for the phase variation observed in MeOPN transferases. However, *C*. *jejuni* has been shown to undergo phase variation at shorter repeats in other genes [41–43] and this is likely the explanation for lack of stoichiometry of fructose.

The CPS of IA3902 appear to be structurally the same as HS1 but with the presence of a MeOPN residue in the 6 position of the galactose, although there is indirect evidence that HS1 also contains the same structure (40). A recent study showed that an encapsulated HS2 strain expressing the *porA* gene from IA3902 became capable of inducing abortion in a guinea pig model [29]. This suggests that there is nothing unusual about the CPS of IA3902 but that the presence of any CPS conferring resistance to complement mediated killing was sufficient. These data are consistent with the observations made here.

MeOPN modifications have been previously shown to play a critical role in conferring resistance to complement-mediated killing in the HS23/36 strain, 81–176 [13]. In this case it appears that the modifications contribute by limiting the ability of pre-existing antibodies in human sera from binding to the basic trisaccharide repeat of the HS23/36 strain [21]. In the HS1 strain, MeOPN also appears to contribute to serum resistance since the *mpnC* mutant was as sensitive as a *kpsM* mutant. In the case of the HS44 type strain, which expresses two CPS, loss of MeOPN on the heptose containing CPS reduced serum resistance, but some level of resistance remains. In contrast, the *kpsM* mutant is very sensitive to killing. These data suggest that the heptose CPS is a primary structure providing serum resistance.

The MeOPN modifications were shown to be immunodominant on conjugates composed of the HS23/36 CPS. It remains to be seen if MeOPN modifications would be equally important to the immunogenicity of HS1 complex conjugates, but it would not be surprising given the uniqueness of this structure. It does appear, however, that a single vaccine based on the main structure of the HS1 type strain should be effective against most members of the complex. We are currently developing HS1 based vaccines in order to confirm this prediction.

## Supporting information

S1 Fig (JPG)
