# Supplementary figures and images for: Relationships of capsular polysaccharides belonging to Campylobacter jejuni HS1 serotype complex
Source: PLoS One. 2021 Feb 23;16(2):e0247305. doi: 10.1371/journal.pone.0247305 (PMC7901785; doi:10.1371/journal.pone.0247305)

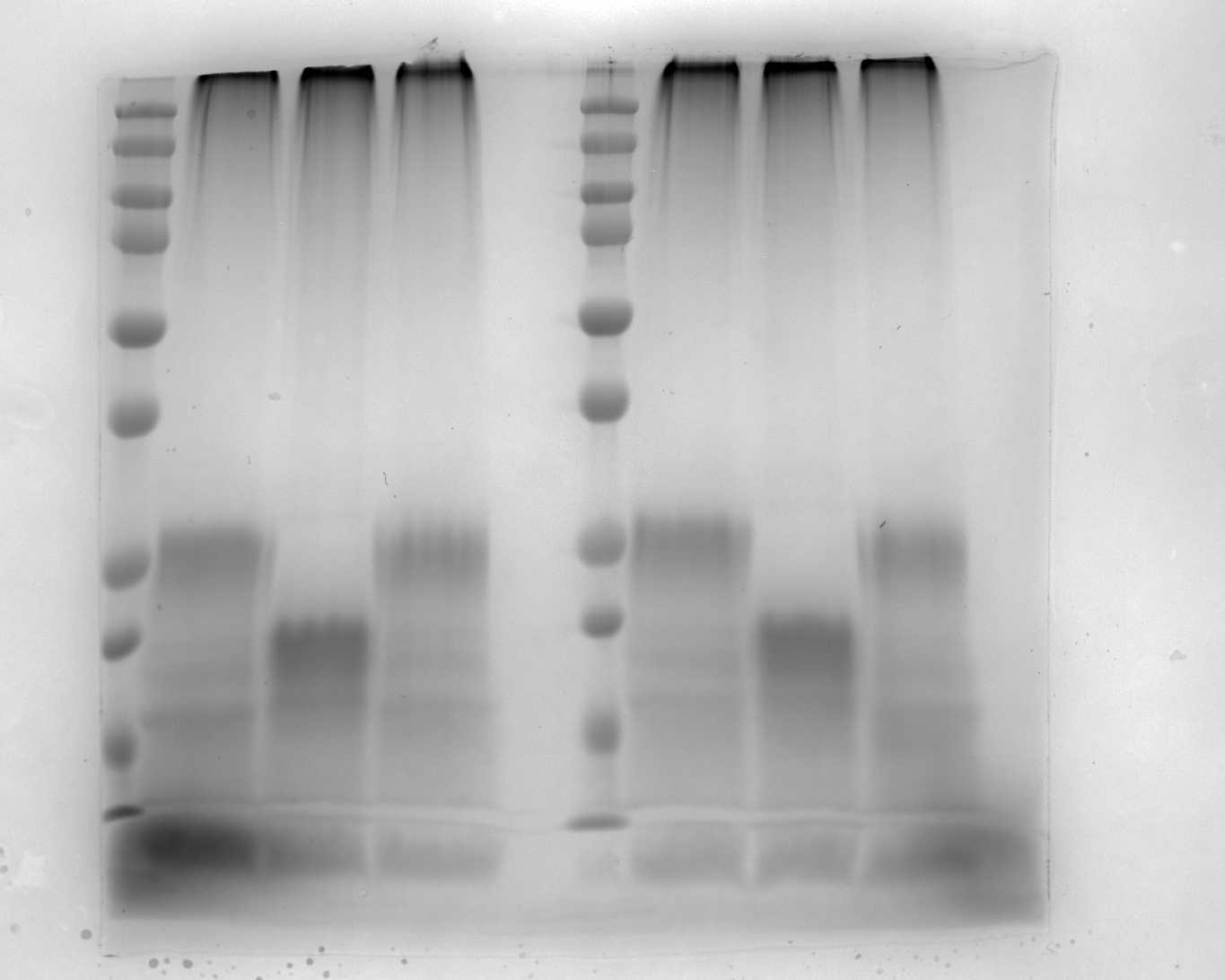

Supplement: S1 Fig — (JPG) [file pone.0247305.s001.jpg]
